# Lessons from Belgium: Physician-assisted dying for (neuro)psychiatric suffering after 23 years of Belgian euthanasia legislation

**DOI:** 10.1192/j.eurpsy.2025.195

**Published:** 2025-08-26

**Authors:** J. Vandenberghe

**Affiliations:** 1 Dpt Neurosciences, Psychiatry Research Group, University KU Leuven; 2 Adult Psychiatry, University Psychiatric Center KU Leuven, Leuven, Belgium

## Abstract

**Abstract:**

In 2002, the euthanasia law was voted in Belgian parliament, depenalising physician-assisted dying under certain conditions for irremediable physical or mental suffering caused by an incurable condition for which all therapeutic options have been exhausted. The euthanasia request needs to be repeated, well-considered and voluntary and the patient should be competent. If the patient is not in a terminal condition, there should be at least one month between the written request and the euthanasia and three independent physicians have to be involved in the evaluation.

Psychiatric suffering was not excluded in the law, but there is discussion whether the possibility of psychiatric euthanasia was intended by the legislator. In the first years after the euthanasia law, psychiatric euthanasia was limited to a few cases, but then rose to a mean of 25 cases per year. There was a peak in 2013 of 54 cases, but after 2013 there was no more increasing trend.

In 2017, the Flemish Association for Psychiatry issued an advice regarding due diligence in psychiatric euthanasia. Controversy regarding psychiatric euthanasia kept stirring the public debate, especially after one court case in which a psychiatrist and general physician were accused and acquitted after a psychiatric euthanasia.

Another prominent topic in the public debate in Belgium is broadening the euthanasia law for advanced dementia based on an advance directive. Now euthanasia is only possible in the earlier stages of neurodegenerative disease, when competence is still sufficiently intact.

**Disclosure of Interest:**

None Declared

